# Adverse effects of homeopathy: a systematic review of published case reports and case series – comment by Tournier *et al*

**DOI:** 10.1111/ijcp.12138

**Published:** 2013-03-24

**Authors:** A Tournier, E R Roberts, P Viksveen

**Affiliations:** Homeopathy Research InstituteLondon, UK

## Abstract

**Linked Comment:** Jackson. *Int J Clin Pract* 2013; 67: 385.

**Linked Comment:** Walach *et al*. *Int J Clin Pract* 2013; 67: 385–6.

**Linked Comment:** Posadzki and Ernst. *Int J Clin Pract* 2013; 67: 386–7.

**Linked Comment:** Grimes. *Int J Clin Pract* 2013; 67: 387.

**Linked Comment:** Posadzki and Ernst. *Int J Clin Pract* 2013; 67: 389.

To the Editor:

We write to express grave concern about the recent article by Posadzki et al. 1 in which the authors claim to have identified 1159 patients who have experienced mild-to-severe adverse effects (AE) caused either directly or indirectly by homeopathic treatment, including four fatalities.

Our concern about this systematic review is not that it highlights the potential for AE from homeopathy, as it would be most peculiar for any medical intervention to be entirely harm-free. Rather, on scrutiny of the article, we have discovered that the reporting of published cases, as well as the methods and analyses applied by the authors are seriously flawed, leading to unreliable conclusions about the relative safety of homeopathy.

Detailed examination of the 37 original articles cited by Posadzki and colleagues has uncovered numerous striking errors. As there are too many to mention in this comment, we draw your attention to just some examples of our key concerns below.

## Misreporting

In reporting on four cases of AEs published by Ibsen et al., Posadzki and colleagues categorise these as ‘likely’ to have been caused by homeopathy^[23]^. However, there is actually no reference made to homeopathy whatsoever in the Ibsen article. The word ‘homeopathy’ only appeared in the English abstract as an incorrect translation of the term ‘alternative treatment’.

Of even greater concern is the reporting of a case report by Geukens^[20]^ of cure by homeopathy, which has been reported by Posadzki et al. as a case of homeopathy causing ‘heart disease and bladder cancer’. In actual fact, the patient was cured from his initial symptoms of vertigo and heart disease using homeopathic medicines; he then presented 7 years later with cancer of the bladder. It is difficult to see how the causality of the cancer could be attributed to the successful treatment of the heart-condition. The cancer was subsequently treated using conventional treatment, the side-effects of which were successfully dealt with using homeopathy. The patient recovered, with no further complaint. How does one end up with causality ‘Almost certain’ for homeopathy in a clear case were homeopathy was instrumental in providing cure?

Another striking example is a case of DRESS (Drug Rash with Eosinophilia and Systemic Symptoms) presented by Bernez et al.^[10]^. In this case, a homeopathic sleeping pill was taken on two occasions without any AE (4 months and 3 weeks before DRESS occurred). The original authors (a team of dermatologists at the University of Tours, France) state very clearly in their conclusions that they do not believe homeopathy was causative in this case. Yet Posadzki et al. report the causality caused by homeopathy as ‘Certain’.

Such instances of significant divergence between the conclusions of the original authors as published in their case reports and Posadzki et al.'s reporting of them, cast a heavy shadow on the scientific validity of the whole review (Data S1).

## Differentiating between ordinary standards of homeopathic care and clinical negligence

When assessing the safety of a medical intervention it is usual to differentiate between non-preventable AEs and those which are preventable with ordinary standards of care 2 i.e. cases of clinical negligence. Posadzki et al. have failed to make any such distinction. The four deaths they report as being caused by homeopathy involve either misprescribing of poisonous substances^[8,32]^ or failure to refer the patient for essential conventional medical treatment^[41,26]^. As these cases contravene what is considered standard homeopathic care, they should be classified as cases of clinical negligence 3 (Data S1).

## Inclusion of non-homeopathic cases

Failure to clearly define ‘homeopathy’ or a ‘homeopathic medicinal product’ at the outset of this review has led to inclusion of multiple cases of misprescribing of poisons as well as cases which are simply not homeopathy at all. For example, a case of AEs caused by Rhus toxicodendron tincture^[34]^ belongs in a review of herbal medicine, not homeopathy, as does the report of a pharmacist who self-administered a tincture of the poisonous plant Aconite^[22]^.

## Inaccuracies

Even at a glance, the Posadzki et al. article does not inspire confidence in its reporting standards. In their Abstract they state 38 reports met their eligibility criteria, whereas on the same page in the results section they state that 35 reports met their eligibility criteria, yet their results tables actually include 37 reports. Two indirect AEs were also misclassified as direct AEs (Data S1).

The authors have also shown a lack of consistency in their decision-making processes regarding inclusion/exclusion of data. For example, 1070 of the 1159 cases identified by Posadzki et al. come from a single article reporting calls to a toxicological information centre. These comprised calls for information (e.g. following inadvertent ingestion of a homeopathic remedy) some of which lead to actual AE cases with a maximum severity of ‘minor’ or ‘mild’^[38]^. As the article by Zuzak et al.^[40]^ presented 2143 similar cases, it is unclear why these were omitted.

Whilst it is beyond the scope of this comment to present a full re-analysis of the data, our examination of the original literature has raised many important issues worthy of further investigation e.g. the need to clearly define homeopathic medicinal products. We therefore recommend that a new review be carried out using rigorous methods to produce a reliable study of the safety of homeopathy.

As clinicians look to systematic reviews on safety to inform their clinical decision-making, any claims that an intervention carries the risk of causing serious harm or death must be based on research carried out to the highest of standards, both in terms of process and accuracy.

Sadly the quality of this review by Posadzki et al. falls short of this standard by such a large margin, that at best the authors’ results are unreliable, and at worst we must consider whether the degree of inaccuracy is such that retraction of this article becomes necessary to preserve the quality of the peer-reviewed literature.

## Supporting Information

**Data S1.** Response to Posadzki et al. 2012.

## References

[1] Posadzki P, Alotaibi A, Ernst E (2012). Adverse effects of homeopathy: a systematic review of published case reports and case series. Int J Clin Pract.

[2] Vincent C (2001). Adverse events in British hospitals: preliminary retrospective record review. BMJ.

[3] Oyebode F (2006). Clinical errors and medical negligence. Adv Psychiatr Treat.

