# Supplementary material for: Adverse effects of homeopathy: a systematic review of published case reports and case series – comment by Tournier et al
Source: Int J Clin Pract. 2013 Mar 24;67(4):388–9. doi: 10.1111/ijcp.12138 (PMC3644880; doi:10.1111/ijcp.12138)
Supplement: Supplementary file 1 [file ijcp0067-0388-SD1.docx]

Response to Posadzki *et al.* 2012 – Supplementary Material

Contents

[Further comments on the four instances with extreme divergence of interpretation 1](#_Toc345665657)

[Further comments on defining clinical negligence 3](#_Toc345665658)

[Further comments on the four deaths reported by Posadzki *et al*. 3](#_Toc345665659)

[Further comments on the two cases of misclassification 4](#_Toc345665660)

# Further comments on the four instances with extreme divergence of interpretation

We find it deeply concerning that in one instance^[10]^ the assessment of causality provided by Posadzki *et al.* diverges so significantly, and without argumentation, from the clearly expressed opinion of the original authors. It is hard to see how this can be justified, considering that the original authors were experts in the field with first-hand knowledge of the case. In a further two instances^[20,13]^ it is difficult to see how the cases could be construed to be AEs caused by homeopathy. In the remaining case^[23]^ one has to wonder whether Posadzki and colleagues actually read the original case report at all.

**Instance 1: Bernez *et al*. 2008^[10]^**

**Posadzki primary classification of AE:** ‘Direct’ **Posadzki AE reported:** ‘DRESS syndrome, severe pulmonary involvement’
**Posadzki assessment of causality:** ‘Certain’
**Our assessment of causality using WHO criteria:** Unlikely*
*‘Event or laboratory abnormality, with a time to drug intake that makes a relationship improbably (but not impossible)’ and ‘Disease or other drugs provide plausible explanations’

**Original reference (translation)**: "By definition the hypersensitivity syndrome (DRESS: Drug Reaction with Eosinophilia and Systemic Symptoms) is drug induced. The only drug implicated from a chronological point of view in this observation is the Sedatif PC, with a weak intrinsic accountability considering the single dose taken three weeks before the start of the eruption. As regards the extrinsic, bibliographic, accountability, no case of DRESS has until now been attributed to homeopathy, and taking account of the extreme dilutions of these medicines, such an adverse effect is very unlikely. The accountability is therefore very low.

It is more probable that this is a coincidence and that the cause originates from the taking of a different drug, which the patient had no recollection of or did not want to declare taking or from a non-drug related cause. (p141,col2,l21-36)"

**Comments:**The original conclusions of the paper very clearly indicate that the authors (three specialist medical doctors directly involved in the case from a hospital Dermatology department in Tours, France) did not believe there was any causal link between taking the homeopathic remedy and the reported clinical case, whereas Posadzki *et al*. rate this case a ‘certain’ in terms of homeopathy causality.

**Instance 2: Geukens 2001^[20]^**

**Posadzki primary classification of AE:** ‘Direct’ **Posadzki assessment of causality:** ‘Almost certain’

**Posadzki adverse event reported:** ‘Heart disease and bladder cancer’
**Our assessment of causality using WHO criteria:** Unlikely*
*‘Disease or other drugs provide plausible explanations’

The abstract to this paper reads: “ … Both of the following cases are actually the same patient, presenting with two distinct pathologies – heart disease and cancer of the bladder. The keynote symptom in the first instance, when the patient suffered angina, was remarkable amelioration produced by strenuous exertion, leading to the prescription of Rhus Toxicodendron, which acted thoroughly. Subsequently, the patient developed cancer of the bladder, that occurrence suggesting that even after a correct homeopathic remedy has been prescribed, a patient may subsequently contract a cancerous condition. The symptoms of the patient then – burning dysuria, involuntary stools, and dreams of dancing - led to the prescription of Gambogia, with very gratifying results.”

C**omments:** Clearly, these two cases involve homeopathic treatment of two separate diseases using homeopathy, at different points in the patient’s medical history. It is extremely hard to see how these cases could be considered as being caused by homeopathy, although Posadzki *et al.* rate causality as ‘almost certain’.

**Instance 3: Colin 2006^[13]^**

**Posadzki primary classification of AE:** ‘Substitution of conventional care with homeopathy’ **Posadzki assessment of causality:** ‘Likely’

**Posadzki condition reported:** ‘Deterioration of pulmonary allergy’

**Our assessment of causality using WHO criteria:** Unlikely*
*‘Disease or other drugs provide plausible explanations’

This case series reports on 147 cases of respiratory allergies treated using homeopathy and attests to the fact that homeopathy is a useful treatment option in such cases. The prescriber – who is both a General Practitioner (GP) and homeopath – discusses fully the fact that patients are provided with conventional treatment where indicated. In two of the 147 cases reported the condition of the patient deteriorated. Posadzki *et al.* classify these two cases as indirect AEs due to substitution of homeopathy for conventional care, yet clearly, conventional care was provided throughout. It is difficult to see how Posadzki *et al.* could consider these two cases as being ‘likely’ due to the homeopathy.

**Instance 4: Ibsen 1987^[23]^**

**Posadzki primary classification of AE:** ‘Substitution of conventional care with homeopathy’
**Posadzki assessment of causality:** ‘Likely’

**Posadzki condition reported:** ‘Severe aggravation of atopic dermatitis’

**Our assessment:** Case has nothing to do with homeopathy

Posadzki *et al.* report four cases of substitution of conventional care from this reference, however on inspection of the original paper, we see there is actually NO reference made to homeopathy what so ever. The word homeopathy only appeared in the English version of the title as an incorrect translation of the term 'alternative medicine', yet this does not deter Posadzki and colleagues from reporting these cases as ‘likely’ caused by homeopathy.

# Further comments on defining clinical negligence

Three conditions must be met in a case of medical negligence, one of which is failure of the doctor to provide the standard of care needed to fulfil this duty^1^. In the UK, the standard of care is primarily determined by the Bolam test^2^.

The Bolam test recognises that there might be several schools of thought regarding proper medical management, which allows medical practitioners to rebut a charge of negligence if they can show that they acted in accordance with practice approved by a body of other responsible practitioners.

In the four cases of deaths reported by Posadzki *et al*.^[8, 32, 26, 41]^ we argue that the care provided would not be considered acceptable as judged by a body of other responsible homeopathic practitioners and are therefore cases of clinical negligence (see below for detail of cases).

# Further comments on the four deaths reported by Posadzki *et al*.

We argue that the four deaths Posadzki’s team report as being caused by homeopathy are in fact cases of clinical negligence i.e. the care provided would not meet the standard of an ordinary and competent qualified homeopath acting responsibly (see previous section).

Two are cases of inappropriate prescribing: In Spain a prescription containing material doses of two poisonous herbs (*Nux-vomica* and *Rhus-tox)* caused death from acute pancreatitis^[8]^, and in India a homeopath self-prescribed a low dilution 'arsenic-containing homeopathic preparation' for 3 years to improve his sexual performance, and contracted cancer and died 50 years later^[32]^. Such poisonous preparations would not be available from EU pharmacies without a medical doctor’s prescription^3^ and would certainly not be prescribed by an ‘ordinary and competent qualified homeopath acting responsibly’ due to their clearly toxic nature.

In the remaining two cases the person prescribing failed to provide standard care. In both instances, one in Israel^[26]^ and one in Australia^[41]^, the prescriber failed to acknowledge that the homeopathic treatment given was ineffective and, despite increasing severity of the patient’s clinical condition, the appropriate referral for essential conventional treatment was not made. It should also be noted that in the Australian case, the prescriber was the child’s father – an unqualified, unregistered ‘homeopath’. The judge who presided over the court case investigating this child’s death found that, ‘…This case does not concern the failure of homeopathy. Rather, it concerns the gross criminal negligence of two parents who failed to ensure that their infant daughter received necessary and appropriate medical care and attention for a treatable condition”.^4^

# Further comments on the two cases of misclassification

**Instance 1: Goodyear & Harper 1990^[21]^**

**Posadzki primary classification of AE:** ‘Direct’ **Posadzki assessment of causality:** ‘Almost certain’

**Posadzki adverse event reported:** ‘Hyponatraemia, hypoalbuminaemia, erythaema and limb oedema’
**Our assessment of causality:** Substitution of conventional care with homeopathy

In the discussion of the case the original authors state: “While homeopathic medicine has relieved some diseases, in our opinion there is little evidence to suggest it is helpful in atopic eczema. Thus caution is needed in the use of homeopathic medicines as the sole treatment for this condition. Severe exacerbations should be recognised and treated early by conventional medicines to avoid the risk of potentially life threatening complications.”

**Comments:** This is not a case caused by homeopathy but a case of substitution of conventional care with homeopathy. Therefore it should be classified as an indirect AE, yet Posadzki *et al.* classify this case as a direct AE with ‘almost certain’ causality from homeopathy.

**Instance 2: Kuenzli et al. 2004^[25]^**

**Posadzki primary classification of AE:** ‘Direct’ **Posadzki assessment of causality:** ‘Likely’

**Posadzki adverse event reported:** ‘Bullous pemphigoid, severe asthaenia’
**Our assessment of causality:** Substitution of conventional care with homeopathy

The abstract of the original paper reads: “… While the role of homeopathy in triggering the disease remains unclear, our observation attests to the potential life-threatening course of childhood BP in instances where the appropriate treatment is withheld.”

In the discussion the original authors state: “Although in the present case no conclusions about the role of homeopathy in the triggering of BP can be made, clinicians should be aware of the potential side effects associated with homeopathy, the indications of which should be carefully evaluated according to the clinical settings as illustrated in this case.”

**Comments:** The original authors (Dept Dermatology, University Hospital, Geneva) clearly indicate that the causation of this case cannot be attributed to homeopathy. This is a case of substitution of conventional care with homeopathy, therefore it should be classified as an indirect AE, yet Posadzki *et al.* classify this case as a direct AE with ‘Likely’ causality from homeopathy.

**Acknowledgements**

Many thanks to Kate Chatfield and Dr Robert Mathie for fruitful discussions and to Stephanie Arnberg-Bengtsson, Dr Emma del Carmen Macias Cortes, Rivka Klein de Graaf, Yanai Lev-or, Christine Liebing-Gabel, Ulrike Kessler, Risto Taipani Raihala, Dr Celia Steiman and Ingrid Wawra for help with translation of non-English articles.

**References**

References in square brackets refer to references from the original Posadzki *et al.* paper^5^.

1. Oyebode, F. Clinical errors and medical negligence. *APT* **12**, 221–227 (2006).

2. Bolam v Friern Hospital Management Committee [1957] 1 WLR 582.

3. Directive 2001/83/EC of the European Parliament and of the Council of 6 November 2001 on the Community code relating to medicinal products for human use. *Official Journal of the European Communities* **L311**, 67-128 (2001).

4. Freckelton, Ian. Complementary health issues. Death by homoeopathy: Issues for civil, criminal and coronial law and for health service policy. *JLM*, **19**, 454-478 (2012).

5. Posadzki, P., Alotaibi, A. & Ernst, E. Adverse effects of homeopathy: a systematic review of published case reports and case series. *Int. J. Clin. Pract.* **66**, 1178–1188 (2012).
